# The role of radiotherapy in MPNST and the impact of NF1 status on outcomes: Insights from a multicenter cohort study

**DOI:** 10.1093/neuonc/noaf186

**Published:** 2025-08-09

**Authors:** Christianne Y M N Jansma, Dirk J Grünhagen, Uta E Flucke, Willem-Bart M Slooff, Thijs van Dalen, Lukas B Been, Han J Bonenkamp, Monique H M E Anten, Martinus P G Broen, Marc H A Bemelmans, Jos A M Bramer, Gerard R Schaap, Arthur J Kievit, Winan J van Houdt, Jos van der Hage, Michiel A J van de Sande, Courtney Pendleton, Robert J Spinner, J Henk Coert, Cornelis Verhoef, Walter Taal, Enrico Martin

**Affiliations:** Department of Plastic and Reconstructive Surgery, University Medical Center Utrecht, Utrecht, The Netherlands; Department of Surgical Oncology and Gastrointestinal Surgery, Erasmus MC Cancer Institute, Rotterdam, The Netherlands; Department of Surgical Oncology and Gastrointestinal Surgery, Erasmus MC Cancer Institute, Rotterdam, The Netherlands; Department of Pathology, Radboud University Medical Center, Nijmegen, The Netherlands; Department of Neurosurgery, University Medical Center Utrecht, Utrecht, The Netherlands; Department of Surgical Oncology and Gastrointestinal Surgery, Erasmus MC Cancer Institute, Rotterdam, The Netherlands; Department of Surgical Oncology, University Medical Center Groningen, Groningen, The Netherlands; Department of Surgical Oncology, Radboud University Medical Center, Nijmegen, The Netherlands; Department of Neurology, Maastricht University Medical Center+, Maastricht, The Netherlands; Department of Neurology, Maastricht University Medical Center+, Maastricht, The Netherlands; Department of Surgical Oncology, Maastricht University Medical Center+, Maastricht, The Netherlands; Department of Orthopedic Surgery, Amsterdam University Medical Center, Amsterdam, The Netherlands; Department of Orthopedic Surgery, Amsterdam University Medical Center, Amsterdam, The Netherlands; Department of Orthopedic Surgery, Amsterdam University Medical Center, Amsterdam, The Netherlands; Department of Surgical Oncology, The Netherlands Cancer Institute, Amsterdam, The Netherlands; Department of Surgical Oncology, Leiden University Medical Center, Leiden, The Netherlands; Department of Orthopedic Oncology, Leiden University Medical Center, Leiden, The Netherlands; Department of Neurosurgery, Mayo Clinic, Rochester, Minnesota, USA; Department of Neurosurgery, Mayo Clinic, Rochester, Minnesota, USA; Department of Plastic and Reconstructive Surgery, University Medical Center Utrecht, Utrecht, The Netherlands; Department of Surgical Oncology and Gastrointestinal Surgery, Erasmus MC Cancer Institute, Rotterdam, The Netherlands; Department of Neurology, Erasmus MC Cancer Institute, Rotterdam, The Netherlands; Department of Plastic and Reconstructive Surgery, University Medical Center Utrecht, Utrecht, The Netherlands

**Keywords:** local recurrence, malignant peripheral nerve sheath tumors, neurofibromatosis type 1, radiotherapy, soft tissue sarcoma

## Abstract

**Background:**

Malignant peripheral nerve sheath tumors (MPNSTs) are rare, aggressive sarcomas, with 40% associated with neurofibromatosis type 1 (NF1). Surgical excision is the main treatment for localized disease; however, local recurrence (LR) remains high. Radiotherapy (RTx) is increasingly used to enhance local control in STS, but its use remains controversial due to the potential for increased major wound complications and an increased risk of secondary malignancies in NF1 patients. This study investigated the use and impact of RTx on local control in MPNSTs, particularly in the NF1 setting.

**Methods:**

Surgically treated primary MPNSTs from 1988 to 2019 in the MONACO multicenter cohort were included. Differences in demographics, treatment, and RTx use between NF1 and non-NF1 cases were examined. Factors influencing RTx administration and LR were assessed via multivariable analyses.

**Results:**

Among 499 patients (33.1% NF1), 143 (28.7%) experienced LRs. Radiotherapy was administered to 56.3% of patients (57.0% in the NF1 group), with 27.3% receiving neoadjuvant and 72.7% adjuvant RTx. RTx was administered significantly more often to high-grade and extremity tumors. While RTx did not affect overall survival, it reduced LR risk in sporadic cases (hazard ratio [HR] 0.530; 95% confidence interval [CI], 0.354–0.793) but not in the NF1 subgroup (HR 1.00; 95% CI, 0.545–1.85). In NF1 patients, a microscopically positive margin (R1) was the only risk factor for LR development (HR 2.1; 95% CI, 1.19–3.79).

**Conclusions:**

RTx is frequently used in the treatment of MPNSTs, regardless of NF1 status. While it may affect the LR rate in sporadic cases, its impact on NF1 patients is less clear.

**Key Points**

• Radiotherapy (RTx) was associated with reduced local recurrence (LR) risk in sporadic malignant peripheral nerve sheath tumors (MPNSTs); this association was not present in neurofibromatosis type 1 (NF1)-associated MPNST.
• In NF1 patients, positive surgical marigins (R1) are a risk factor for LR.
• Tailored treatment is needed for MPNSTs, especially in NF1 cases.

**Importance of the Study**

Malignant peripheral nerve sheath tumors (MPNSTs) are aggressive sarcomas often associated with neurofibromatosis type 1 (NF1), with high recurrence rates. This multicenter cohort study evaluates the role of RTx in managing MPNSTs, highlighting its limited effect in NF1-associated cases. Compared to prior studies, this research provides a comprehensive evaluation of RTx efficacy in a large patient population. The findings underscore the need for personalized treatment strategies based on tumor characteristics and patient-specific risk factors. Moreover, this study emphasizes the importance of further research into the molecular differences between NF1-associated and sporadic MPNSTs, aiming to refine treatment protocols and improve outcomes. These insights have direct clinical relevance, guiding future therapeutic decisions and potentially reducing unnecessary RTx exposure in NF1 patients.

Malignant peripheral nerve sheath tumors (MPNSTs) are rare and aggressive soft-tissue sarcomas (STS), with 25%–50% of cases occurring in individuals with neurofibromatosis type 1 (NF1).^1,2^ NF1 is an autosomal-dominant genetic disorder in which patients develop various tumor types, including superficial and deep neurofibromas.^3^ The incidence of MPNSTs in NF1 patients is 1 in 2000, compared to 1 in 100 000 in the general population, with a lifetime risk of 8%–13% in NF1 patients.^2–6^ Most NF-1-associated MPNSTs arise from pre-existing plexiform neurofibromas.^7^ Additionally, MPNSTs may also develop sporadically or as a result of radiation exposure in individuals without NF1.^3,7^ Surgical resection with negative margins is the primary treatment for localized MPNSTs, yet recurrence rates, both local and distant, remain high, even after aggressive surgical intervention.^7,8^ To improve local control, perioperative radiotherapy (RTx) is commonly employed.^9^ However, its effectiveness, particularly in NF1-associated MPNSTs compared to sporadic cases, remains inadequately studied.^10^ Clinical guidelines, such as those from the European Society for Medical Oncology (ESMO), recommend individualized treatment strategies based on tumor characteristics, including the use of RTx for high-grade tumors, larger tumors, or in cases with positive surgical margins.^11^ However, in practice, NF1-associated MPNSTs are frequently treated similarly to sporadic cases, even though differences in tumor biology and response to therapy may exist.^12^ While RTx is often employed with the expectation of improving clinical outcomes, its efficacy in NF1-related tumors compared to non-NF1 MPNSTs remains uncertain and warrants further investigation.^12^

Therefore, the primary aim of this project is to evaluate the use of RTx and impact on local recurrence (LR) in MPNST patients, particularly those with NF1.

## Materials and Methods

### Patient Population

A retrospective cohort investigation, the MONACO study, was conducted across 9 sarcoma centers in the Netherlands and the Mayo Clinic following the approval of institutional review boards at each participating center. This study included patients who underwent surgical treatment for their primary tumor and were diagnosed with pathologically confirmed primary MPNST between 1988 and 2019. Diagnosis for all patients adhered to the World Health Organization’s soft tissue and bone tumor classification guidelines.^13^ Patients whose pathological reports or diagnoses were uncertain based on available information during follow-up were not considered for inclusion in the study. Furthermore, patients who presented with LRs who had previously undergone resection elsewhere were also excluded.

### Covariates

Covariates obtained from the medical records for analysis included patient demographics, tumor characteristics, treatment details, and survival-related data. An LR referred to the initial radiological or pathological sign of tumor recurrence at the primary tumor site. Age was determined as the patient’s age at the time of diagnosis. To assess the patients’ physical status, the American Society of Anesthesiologists classification system was employed.^14^ A tumor was classified as NF1-associated based on a confirmed genetic test identifying an NF1 mutation or through clinical assessment.^15^ Tumor size was determined by measuring the largest diameter of the tumor mass, using either imaging techniques or pathology reports. Pathological tumor size was used when available; otherwise, radiologic measurements were used. We could not systematically verify if assessment methods were balanced between groups. Tumor grade was classified into low-grade (grade 1) or high-grade (grade 2 or 3) based on the grading system established by the Fédération Nationale des Centres de Lutte Contre le Cancer (FNCLCC).^16^ Tumors originating below or within the deep fascia were classified as deep-seated. Information on NF1 status was extracted from pathology reports, explicitly stated or inferred from prior reports on plexiform neurofibroma resections or the presence of 2 or more neurofibromas. Surgical margins were classified into three categories with R0 (indicating microscopically negative with no presence of tumor cells in surgical borders), R1 (microscopically positive), and R2 (macroscopically positive). Tumor site was classified as extremity, central (thorax, abdomen, pelvis, retroperitoneal), or head and neck. Triton status was determined by extracting information from pathological reports, specifically when it was explicitly mentioned in the report or when there was a report of MPNST with rhabdomyoblastic differentiation. MPNST occurring in a previously irradiated area that overlapped with the primary tumor site was defined as RTx-associated MPNST. The study’s main endpoints included the use of RTx and the development of an LR in both NF-1 and non-NF1 groups.

### Statistical Analysis

All statistical analyses were conducted using R software (version 4.2.2). Baseline characteristics and treatment modalities were compared between all patients with and without RTx administration. This comparison was also performed in a subset of NF1 patients. Overall survival (OS) was defined as the time from definitive surgery to death or the date of last follow-up for both NF1 and non-NF1 patients. Estimated median survival was calculated using the Kaplan–Meier method for several covariates of interest. Univariable and multivariable logistic regression analyses were performed in all patients to identify risk factors associated with RTx utilization. Multivariable Cox Proportional Hazards models were used to estimate the effect of several covariates on the development of LR1 in NF1 patients, considering death as a competing risk.

Candidate predictors were selected based on clinical knowledge and existing literature. Univariable and multivariable analyses with 95% confidence intervals (CIs) were used to assess the impact of covariates on outcomes. For multivariable models, variables with a *P*-value <.3 or <.4 in the univariable analysis were included, depending on the model criteria. Additionally, multivariable models were refined using backward selection to improve model fit and identify the most significant predictors. Proportional hazards were evaluated visually using Schoenfeld residuals. Missing data were addressed through multiple imputations (*m* = 20), with pooled estimates following Rubin’s rules.^17^ A *P*-value ≤.05 was considered statistically significant. Results from the Cox proportional hazards models were expressed as hazard ratios (HRs) with 95% CI. All statistical tests were 2-sided.

## Results

### Patient Selection and Study Population

**Figure 1.**
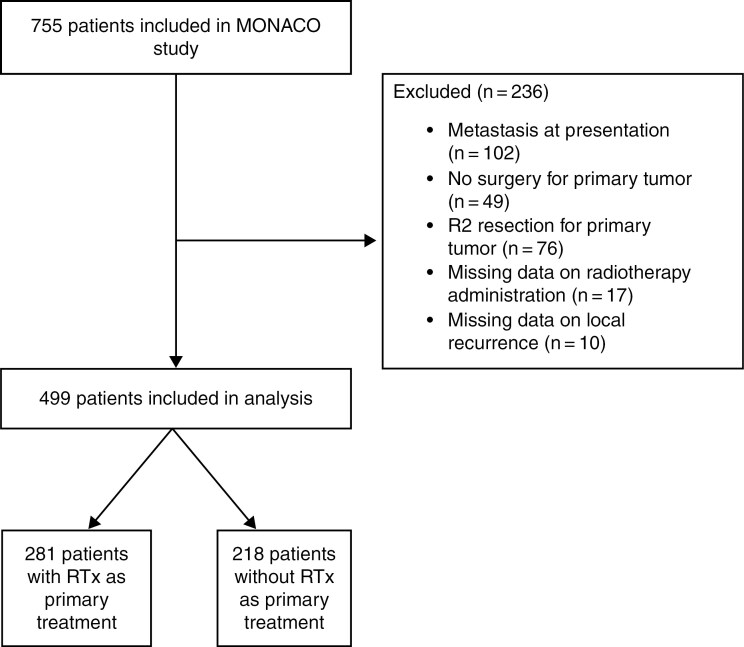
Consort flow diagram. A schematic representation of the study inclusion process, illustrating patient selection and exclusion criteria. *N* = number of patients included at each stage.

A total of 755 patients were included in the MONACO database. Patients were excluded if they presented with metastasis at diagnosis (*n* = 102), were not treated surgically for the primary tumor (*n* = 49), underwent R2 resection (*n* = 76), or had missing data on radiation administration (*n* = 17) or presented with LR (*n* = 10) (Figure 1). This left 499 patients eligible for analysis, of whom 281 (56.3%) received RTx as part of their primary treatment. The clinical and tumor characteristics of all patients are detailed in Table 1. The median follow-up time was 60.5 months (mean = 86.8 months, SD = 78.8 months, range = 1 day to 333 months). Radiotherapy was more commonly administered in patients with larger and high-grade tumors, with a median tumor size of 69 mm (IQR 46–104) compared to 59 mm (IQR 40–90.2) in patients who did not receive RTx (*P* = 0.018). Additionally, extremity tumors were more common among patients who received RTx. Furthermore, we identified a subset of 165 patients with NF1, among whom 57.0% (94 patients) received RTx. Similar trends in indication for RTx were seen among NF1 patients (Supplementary Table 1). Importantly, there was no significant difference in RTx utilization between patients with sporadic tumors and those with NF1, as confirmed in both univariate and multivariable analyses (Table 2). In the sporadic MPNST cohort, the median neoadjuvant RTx dose was 50 Gy (range: 30–56.25 Gy) with a range of 17–30 fractions, and the median adjuvant RTx dose was 60 Gy (range: 27–95 Gy) with a range of 15–38 fractions. Similar dose and fraction distributions were observed in the NF1-associated cohort, with a median neoadjuvant dose of 50 Gy (range: 45–56.25 Gy; 17–30 fractions) and a median adjuvant dose of 60 Gy (range: 27–75 Gy; 15–38 fractions), indicating no meaningful difference in administered RTx doses or fractionation between groups.

**Table 1.** Patient Characteristics of MPNST Patients and RTx Administration

Variable	Overall	No radiotherapy	Any type of radiotherapy	*P*-value
*N*	499	218	281	
Age (years)				
Mean (SD)		42.80 (21.14)	43.52 (18.05)	.683
Male gender	268	113 (51.8%)	155 (55.4%)	.489
NA	1	-	1	
ASA				
I	158	71 (56.8%)	87 (56.9%)	.923
II	106	47 (37.6%)	59 (38.6%)	
III	14	7 (5.6%)	7 (4.6%)	
NA	221	93	128	
NF1				
No	324	144 (67.0%)	180 (65.7%)	.840
Yes	165	71 (33.0%)	94 (34.3%)	
NA	10	3	7	
Tumor size				
<5 cm	130	66 (39.3%)	64 (27.9%)	.043
5–10 cm	162	65 (38.7%)	97 (42.4%)	
>10 cm	105	37 (22.0%)	68 (28.7%)	
NA	102	50	52	
Tumor depth				
Superficial	73	37 (26.8%)	36 (20.7%)	.257
Deep	239	101 (73.2%)	138 (79.3%)	
NA	187	80	107	
Tumor grade				
High grade	281	114 (79.2%)	167 (90.8%)	.005
Low grade	47	30 (20.8%)	17 (9.2%)	
NA	171	74	97	
RTx-associated				
No	448	193 (90.2%)	255 (93.4%)	.258
Yes	51	21 (9.8%)	18 (6.6%)	
NA	12	4	8	
Site of primary tumor				
Head and neck	68	36 (16.5%)	32 (11.4%)	.001
Extremities	249	87 (39.9%)	162 (57.7%)	
Central	180	94 (43.1%)	86 (30.6%)	
Unknown	2	1 (0.5%)	1 (0.4%)	
Surgical margin				
R0	324	148 (67.9%)	176 (62.6%)	.247
R1	140	53 (24.3%)	87 (31.0%)	
Unknown	35	17 (7.8%)	18 (6.4%)	

Abbreviations: *N*, number of; RTx, radiotherapy; NA, not available; NF1, neurofibromatosis type 1; SD, standard deviation.

**Table 2.** Multivariate Logistic Regression on Factors Associated With the Use of Radiotherapy

Variable	OR (CI 95%)	*P*-value
Intercept	0.308 (0.136–0.699)	.007
Tumor size		
<5 cm	1.00	
>5 cm	1.53 (0.906–2.58)	.113
Tumor depth		
Superficial	1.00	
Deep	1.10 (0.607–1.98)	.762
Tumor grade		
Low grade	1.00	
High grade	2.41 (1.20–4.84)	.013
Site of primary tumor		
Head and neck	1.00	
Extremities	1.82 (1.00–3.30)	.050
Central	0.862 (0.457–1.63)	.643
Surgical margin		
R0	1.00	
R1	1.49 (0.977–2.26)	.079

Abbreviations: CI, confidence interval; OR, odds ratio.

### Impact of RTx on Local Recurrence

One hundred and forty-three (28.7%) patients (both sporadic and NF1-associated) developed a LR after surgery for their primary tumor. The median time to an LR was 10.9 months (interquartile range [IQR] 0.6–22.8 months). For the NF1 subgroup, the median time to an LR was 10.5 months (IQR 0.6–21.7 months). Multivariable analysis for risk factors associated with LR (Table 3) revealed that tumor size (>5 cm) and tumor grade (high grade) remained significant risk factors for LR, with HRs of 2.41 (95% CI, 1.22–4.76, *P *= .014) and 2.98 (95% CI, 1.26–7.06, *P* = .016), respectively. R1 margins were strongly associated with increased recurrence risk (HR: 2.93, 95% CI, 1.94–4.41, *P* < .001). In the full cohort, including both NF1-associated and sporadic MPNST patients, the use of RTx was independently associated with a significantly reduced risk of LR (HR 0.530; 95% CI, 0.354–0.793, *P* = .003), while NF1 status, tumor depth, and site of primary tumor did not show significant associations with LR. However, in the NF1 patient subgroup, RTx did not significantly reduce the risk of LR (HR 1.00; 95% CI 0.545–1.85; *P* = .990) (Table 4). In this NF1 subgroup, a total of 165 patients were analyzed, of whom 94 received RTx, and 57 experienced a LR. The only significant risk factor associated with LR risk in NF1 patients was R1 margin status (HR 2.12; 95% CI, 1.19–3.79, *P* = .015). In addition to multivariable analyses, crude LR rates were assessed to further illustrate subgroup differences. Among patients with sporadic MPNSTs, the LR rate was 27.8% in those who did not receive RTx and 23.3% in those who did. In contrast, NF1-associated MPNST patients exhibited higher LR rates overall, with 33.8% in patients without RTx and 35.1% in those who received RTx. The impact of RTx on OS was not significant across the entire cohort (*P* = .50) or within subgroups, including NF1 (*P* = .93) and non-NF1 patients (*P* = .62). Detailed survival analyses are presented in Supplementary Figures 1 and 2.

**Table 3.** Uni- and Multivariate Cox Model on Factors Associated With the Development of an LR in Patients According to the Guideline RTx

	Univariate		Multivariate	
Variable	HR (95%CI)	*P*-value	HR (CI 95%)	*P*-value
Age (per 10 years)	1.05 (0.948–1.15)	.375		
NF1				
No	1.00		1.00	
Yes	1.270 (0.850–1.90)	.246	0.908 (0.589–1.40)	.662
Tumor size				
<5 cm	1.00		1.00	
>5 cm	2.36 (1.29–4.35)	.007	2.41 (1.22–4.76)	.014
Tumor depth				
Superficial	1.00		1.00	
Deep	1.41 (0.801–2.47)	.240	1.21 (0.652–2.24)	.548
Tumor grade				
Low grade	1.00		1.00	
High grade	1.85 (0.804–4.27)	.152	2.98 (1.26–7.06)	.016
Any type of RTx				
No	1.00		1.00	
Yes	0.742 (0.505–1.09)	.131	0.530 (0.354–0.793)	.003
Any type of CTx				
No	1.00			
Yes	0.786 (0.420–1.42)	.453		
Site of primary tumor				
Head and neck	1.00		1.00	
Extremities	1.22 (0.636–2.35)	.547	1.14 (0.563–2.31)	.717
Central	1.54 (0.793–2.97)	.207	1.34 (0.656–2.73)	.427
Surgical margin				
R0	1.00		1.00	
R1	2.32 (1.57–3.44)	<.005	2.93 (1.94–4.41)	<.001

Abbreviations: CI, confidence interval; CTx, chemotherapy; HR, hazard ratio; LR, local recurrence; NF1, neurofibromatosis type 1; RTx, radiotherapy.

**Table 4.** Uni- and Multivariate Cox Model on LR Development in NF1 Patients

	Univariate		Multivariate	
Variable	HR (95% CI)	*P*-value	HR (95% CI)	*P*-value
Tumor size				
<5 cm	1.00		1.00	
>5 cm	1.66 (0.706–3.90)	.253	1.34 (0.548–3.28)	.526
Tumor depth				
Superficial	1.00			
Deep	0.984 (0.449–2.15)	.968		
Tumor grade				
Low grade	1.00		1.00	
High grade	1.76 (0.547–5.68)	.352	1.479 (0.434–5.05)	.538
Any type of RTx				
No	1.00			
Yes	1.09 (0.614–1.92)	.779	1.00 (0.545–1.85)	.990
Site of primary tumor				
Head and neck	1.00		1.00	
Extremities	1.74 (0.603–5.03)	.310	1.56 (0.526–4.65)	.426
Central	1.79 (0.620–5.20)	.286	1.53 (0.517–4.54)	.446
Surgical margin				
R0	1.00		1.00	
R1	2.25 (1.28–3.94)	.007	2.12 (1.19–3.79)	.015

Abbreviations: CI, confidence interval; HR, hazard ratio; LR, local recurrence; RTx, radiotherapy.

## Discussion

In this study, a large cohort of patients with MPNST was analyzed, with just over half (56%) of the patients receiving RTx as part of their primary treatment. Factors such as tumor size, grade, and margin status were associated with an increased risk of LR. In patients with NF1, only positive surgical margins were significantly associated with the development of LR, whereas RTx did not affect LR risk in this group, in contrast to sporadic cases.

For MPNSTs, surgical excision remains the cornerstone of treatment, but achieving complete resection is often challenging in the balance between local controle and limbsalvage. One study, analyzing 784 MPNST, showed that complete resection (R0) was achieved in 66.3% of patients.^18^ As in other STS, RTx is commonly administered to decrease LR rates but has not been shown to correlate with improved survival.^19^ While surgical excision with negative margins remains the most important determinant of local control and potential cure, RTx serves as an adjunct in cases where adequate margins cannot be achieved.^11^ Importantly, in cases where tumors are located near critical neurovascular structures, neoadjuvant RTx can facilitate limb-sparing surgery by reducing tumor size and allowing for less extensive resections, thereby avoiding mutilating procedures such as amputation and preserving function.^20,21^ To date, no research has shown that MPNSTs require a different treatment approach compared to other high-grade STS.^22,23^ Therefore, the treatment guidelines for MPNSTs largely align with those established for general STS.^11,24^ Generally, according to ESMO guidelines, RTx is recommended for tumors with aggressive features such as ≥5 cm in size, high grade, or R1/R2 margins.^11^ In practice, however, RTx use varies significantly between centers, and not all eligible patients receive it.^19^ Differences in institutional protocols, specialist preferences, and biases can lead to inconsistent RTx use, even in high-grade sarcoma cases.^19^ In our cohort, similar variability was seen, with some patients not receiving RTx despite meeting guideline criteria. In our study, we observed that RTx usage patterns in NF1 patients were similar to those in sporadic cases, consistent with literature advocating RTx use in NF1 patients.^25^ It is important to note that there was an imbalance in baseline risk factors between patients who received RTx and those who did not. Specifically, patients treated with RTx were more likely to have larger and higher-grade tumors, which are known negative prognostic factors for LR. This imbalance may have influenced the observed outcomes and should be considered when interpreting the results. The role of RTx in decreasing LR rates remains inconclusive, as varying results have been reported across the literature.^26^ Our LR rate of approximately 30% reflects similar outcomes in other studies combining surgery and RTx.^8,27^ Our findings show that the effect of RTx on LR rates differs between NF1-associated and sporadic MPNSTs. In sporadic cases, RTx was significantly associated with a reduction in LR; however, in NF1-associated MPNSTs, no significant benefit of RTx on LR rates was observed.

A potential explanation for this could be the biological and pathological differences between NF1-associated and sporadic tumors. NF1-associated MPNSTs are often more aggressive and exhibit distinct tumor pathology.^28^ Additionally, in NF1 patients, the neurofibromas surrounding the tumor could have undergone pre-malignant changes, raising the risk of recurrence or secondary tumor formation and potentially limiting the effectiveness of RTx in improving LR rates. Another possible explanation may lie in the underlying molecular and genetic complexity of NF1-associated MPNSTs, which is not yet fully understood.^28^ Although studies have identified recurrent genomic alterations in MPNSTs, no consistent molecular differences between NF1-associated and sporadic MPNSTs have been confirmed.^29–33^ This lack of clarity complicates the prediction of treatment response and suggests that molecular differences may influence treatment outcomes, although specific pathways, such as those involving EGFR or TP53, remain speculative and unproven in their role of RTx resistance.^34–40^ Nevertheless, this finding should be interpreted with caution, as the possibility of a Type II error cannot be excluded. Our study may have been underpowered to detect a significant difference in LR risk reduction with RTx in NF1-associated MPNSTs. Further research with larger cohorts is warranted to validate these results and better define the role of RTx in NF1-associated MPNSTs.

Furthermore, the value of RTx for reducing LR in MPNSTs overall remains ambiguous in the literature.^7,27,41,42^ Many studies do not differentiate between NF1-associated and sporadic MPNSTs when assessing the efficacy of RTx.^8,12,27^ As a result, the reported benefit of RTx may primarily reflect its impact on sporadic tumors, where it has been more consistently associated with reduced LR rates. This conflation of NF1-associated and sporadic cases might mask crucial distinctions in treatment response, particularly given the unique molecular and pathological characteristics of NF1-associated MPNSTs. The lack of subgroup analyses obscures whether NF1-associated MPNSTs respond similarly, leaving a critical gap in understanding the true efficacy of RTx in this specific context. Future studies should prioritize the stratification of outcomes by NF1 status to determine whether and under what conditions RTx offers meaningful benefits for NF1-associated MPNSTs. While our study demonstrates the above findings, it is important to acknowledge that data on the dose of RTx were not available. The efficacy of RTx, particularly in reducing LR, is known to be dose-dependent, and the absence of dose data limits our ability to assess whether variations in dose could have influenced the outcomes observed in our cohort. Therefore, future studies should include detailed dose information to provide further insights into the optimal radiation dose for treating MPNSTs, particularly for those with NF1.

The role of RTx in reducing LR in MPNSTs must also be carefully weighed against potential disadvantages. It has been reported that MPNSTs have been caused by RTx.^43,44^ This concern is especially relevant in NF1 patients, where RTx has been associated with an increased risk of secondary malignancies, including radiation-induced MPNSTs, as documented in several studies.^45,46^ In addition to the risk of secondary MPNSTs in NF1 patients, RTx carries other potential complications that affect both NF1-associated and sporadic cases. For both groups, RTx has been associated with risks such as wound healing complications when administered preoperatively and late-onset radiation toxicities when used postoperatively.^47^ Balancing the benefits of RTx against the risks of secondary malignancies and functional impairments is crucial in treatment planning for both NF1-associated and sporadic MPNST patients. For NF1 patients in particular, careful consideration may be needed due to the elevated risk of secondary malignancies associated with RTx.^46,47^ A multidisciplinary evaluation for both patient groups is essential to ensure optimal care and to tailor treatment strategies to individual needs.^7^

Given the possible absence of significant improvement in LR rates with the use of RTx, it may be advisable to apply a more cautious approach to RTx in NF1 patients, particularly when tumors arise within plexiform neurofibromas. The inherent pre-malignant nature of the surrounding tissue in these cases and the limited impact of RTx on OS and LR outcomes suggest that RTx may offer minimal benefit while posing additional risks.

This multicenter retrospective study has limitations inherent to its design, such as potential selection bias resulting from selective loss of follow-up and missing data. Nevertheless, a multiple imputation technique was employed to reduce this risk of bias. Additionally, the extended study period of 30 years saw the introduction of various new techniques and advancements in radiation therapy delivery, which may have led to variations in treatment standards that could influence the findings. However, due to a lack of detailed data on modality and quality, potential improvements in imaging and RTx techniques over time could not be assessed. Other key limitations include variability in treatment protocols across different centers and the heterogeneity of tumors. Factors such as tumor size, resection margin, malignancy grade, and other often unknown factors likely influenced the decision to administer RTx, which could have affected the primary outcome. Moreover, variations in radiation doses and techniques over time may have influenced treatment effectiveness, an aspect that should be considered when interpreting the results. Another important limitation is the lack of detailed data on the margin extent beyond the R0 versus R1 classification. Precise information on margin width (eg, in millimeters) was not available. This limited our ability to explore more nuanced associations between the degree of surgical clearance and local control. Future studies could investigate whether variations in margin width within the R0 category, such as planned close margins in combination with adjuvant RTx, affect local control outcomes, as this remains a clinically relevant question. Despite these limitations, the large size of this international cohort study on MPNSTs provides valuable insights. To our knowledge, this is the largest cohort study on RTx in MPNSTs, allowing for a more comprehensive analysis of treatment outcomes. The study’s focus on specific histological subtypes, such as NF1, along with detailed clinical and treatment information, enhances our understanding of tumor behavior and aids in tailoring ideal treatment strategies. Furthermore, the multicenter design enhances the generalizability of the findings, as data were collected from multiple sarcoma centers, thus reflecting a diverse patient population and treatment practices.

## Conclusion

In conclusion, this multicenter study demonstrates that while RTx is frequently utilized in the treatment of MPNSTs, its benefit may be limited to certain cases of sporadic MPNSTs, where it is associated with a lower risk of LR. However, this advantage may not extend to the NF1 subpopulation. Despite a substantial number of patients receiving RTx, factors such as tumor size, grade, and surgical margins play a critical role in determining treatment outcomes.

## Supplementary material

Supplementary material is available online at *Neuro-Oncology* (https://academic.oup.com/neuro-oncology).

noaf186_Supplementary_Materials_1

## Data Availability

The data that support the findings of this study are available on request from the corresponding author, upon reasonable request. The data are not publicly available due to information that could compromise the privacy of research participants.

## References

[1] Edizer DT , Özdoğan A, Karaman E, Işıldak H. Report of a case of malignant peripheral nerve sheath tumor of the neck with an early local recurrence. Ear Nose Throat J. 2011;90(10):E1–E3. 10.1177/014556131109001015 22033963

[2] Du P , Zhu J, Zhang ZD, et al Recurrent epithelioid malignant peripheral nerve sheath tumor with neurofibromatosis type 1: a case report and literature review. Oncolo Lett. 2019;18(3):3072–3080. 10.3892/ol.2019.10676 PMC6704279 31452784

[3] James AW , Shurell E, Singh A, Dry SM, Eilber FC. Malignant peripheral nerve sheath tumor. Surg Oncol Clin N Am. 2016;25(4):789–802. 27591499 10.1016/j.soc.2016.05.009

[4] Martin E , Geitenbeek RTJ, Coert JH, et al A Bayesian approach for diagnostic accuracy of malignant peripheral nerve sheath tumors: a systematic review and meta-analysis. Neuro-Oncology. 2021;23(4):557–571. 33326583 10.1093/neuonc/noaa280 PMC8041346

[5] Geitenbeek RTJ , Martin E, Graven LH, et al Diagnostic value of 18F-FDG PET-CT in detecting malignant peripheral nerve sheath tumors among adult and pediatric neurofibromatosis type 1 patients. J Neurooncol. 2022;156(3):559–567. 35025020 10.1007/s11060-021-03936-y PMC8860956

[6] Uusitalo E , Rantanen M, Kallionpää RA, et al Distinctive cancer associations in patients with neurofibromatosis type 1. J Clin Oncol. 2016;34(17):1978–1986. 26926675 10.1200/JCO.2015.65.3576

[7] Kahn J , Gillespie A, Tsokos M, et al Radiation therapy in management of sporadic and neurofibromatosis type 1-associated malignant peripheral nerve sheath tumors. Front Oncol. 2014;4(4):324. 25452937 10.3389/fonc.2014.00324 PMC4233912

[8] Bishop AJ , Zagars GK, Torres KE, et al Malignant peripheral nerve sheath tumors: a single institution’s experience using combined surgery and radiation therapy. Am J Clin Oncol. 2018;41(5):465–470. 27281262 10.1097/COC.0000000000000303 PMC5145780

[9] Sobczuk P , Teterycz P, Czarnecka AM, et al Malignant peripheral nerve sheath tumors–outcomes and prognostic factors based on the reference center experience. Surg Oncol. 2020;35(35):276–284. 32949967 10.1016/j.suronc.2020.09.011

[10] Prudner BC , Ball T, Rathore R, Hirbe AC. Diagnosis and management of malignant peripheral nerve sheath tumors: current practice and future perspectives. Neurooncol. Adv.. 2020;2(Suppl 1):i40–i49. 32642731 10.1093/noajnl/vdz047 PMC7317062

[11] Gronchi A , Miah AB, Dei Tos AP, et al EURACAN and GENTURIS. Electronic address: clinicalguidelines@ esmo. org. Soft tissue and visceral sarcomas: ESMO-EURACAN-GENTURIS Clinical Practice Guidelines for diagnosis, treatment and follow-up. Ann Oncol. 2021;32(11):1348–1365. 34303806 10.1016/j.annonc.2021.07.006

[12] Roohani S , Claßen NM, Ehret F, et al The role of radiotherapy in the management of malignant peripheral nerve sheath tumors: a single-center retrospective cohort study. J Cancer Res Clin Oncol. 2023;149(20):17739–17747. 37924493 10.1007/s00432-023-05449-9 PMC10725397

[13] Jo VY , Fletcher CDM. WHO classification of soft tissue tumours: an update based on the 2013 (4th) edition. Pathology (Phila). 2014;46(2):95–104. 10.1097/PAT.0000000000000050 24378391

[14] Saklad M. Grading of patients for surgical procedures. Paper presented at: The Journal of the American Society of Anesthesiologists. 1941. 2(3) 281–284

[15] Neurofibromatosis. Conference statement. National Institutes of Health Consensus Development Conference. Arch Neurol. 1988;45(5):575–578. 3128965

[16] Coindre J-M. Grading of soft tissue sarcomas: review and update. Arch Pathol Lab Med. 2006;130(10):1448–1453. 17090186 10.5858/2006-130-1448-GOSTSR

[17] Rubin DB. Multiple imputation after 18+ years. J Am Stat Assoc. 1996;91(434):473–489.

[18] Martin E , Coert JH, Flucke UE, et al A nationwide cohort study on treatment and survival in patients with malignant peripheral nerve sheath tumours. Eur J Cancer. 2020;124(124):77–87. 31760312 10.1016/j.ejca.2019.10.014

[19] Martin E , Slooff W-BM, van Houdt WJ, et al Oncological treatment considerations differ across surgical subspecialties treating malignant peripheral nerve sheath tumors: an international survey. Sarcoma. 2020;2020(1):1–10. 10.1155/2020/6406439 PMC7064831 32189989

[20] Seinen JM , Hoekstra HJ. Isolated limb perfusion of soft tissue sarcomas: a comprehensive review of literature. Cancer Treat Rev. 2013;39(6):569–577. 23232098 10.1016/j.ctrv.2012.10.005

[21] Eggermont AMM , Koops HS, Klausner JM, et al Isolated limb perfusion with tumor necrosis factor and melphalan for limb salvage in 186 patients with locally advanced soft tissue extremity sarcomas: the cumulative multicenter European experience. Ann Surg. 1996;224(6):756–765. 8968230 10.1097/00000658-199612000-00011 PMC1235474

[22] Gronchi A , Ferrari S, Quagliuolo V, et al Histotype-tailored neoadjuvant chemotherapy versus standard chemotherapy in patients with high-risk soft-tissue sarcomas (ISG-STS 1001): an international, open-label, randomised, controlled, phase 3, multicentre trial. Lancet Oncol. 2017;18(6):812–822. 28499583 10.1016/S1470-2045(17)30334-0

[23] Callegaro D , Miceli R, Bonvalot S, et al Impact of perioperative chemotherapy and radiotherapy in patients with primary extremity soft tissue sarcoma: retrospective analysis across major histological subtypes and major reference centres. Eur J Cancer (Oxford, England : 1990). 2018;105(105):19–27. 10.1016/j.ejca.2018.09.028 30384013

[24] Von Mehren M , Randall RL, Benjamin RS, et al Soft tissue sarcoma, version 2.2016, NCCN clinical practice guidelines in oncology. J Nat Compr Canc Netw. 2016;14(6):758–786. 10.6004/jnccn.2016.0078 27283169

[25] Carton C , Evans DG, Blanco I, et al; ERN GENTURIS NF1 Tumour Management Guideline Group. ERN GENTURIS tumour surveillance guidelines for individuals with neurofibromatosis type 1. EClinicalMedicine. 2023;56(56):101818. 36684394 10.1016/j.eclinm.2022.101818 PMC9845795

[26] Yang JC , Chang AE, Baker AR, et al Randomized prospective study of the benefit of adjuvant radiation therapy in the treatment of soft tissue sarcomas of the extremity. J Clin Oncol. 1998;16(1):197–203. 9440743 10.1200/JCO.1998.16.1.197

[27] Wong WW , Hirose T, Scheithauer BW, Schild SE, Gunderson LL. Malignant peripheral nerve sheath tumor: analysis of treatment outcome. Int J Radiation Oncol Biol Phys. 1998;42(2):351–360. 10.1016/s0360-3016(98)00223-5 9788415

[28] Kolberg M , Høland M, Ågesen TH, et al Survival meta-analyses for> 1800 malignant peripheral nerve sheath tumor patients with and without neurofibromatosis type 1. Neuro-Oncology. 2013;15(2):135–147. 23161774 10.1093/neuonc/nos287 PMC3548581

[29] Mertens F , Dal CP, De Wever I, et al Cytogenetic characterization of peripheral nerve sheath tumours: a report of the CHAMP study group. J Pathol. 2000;190(1):31–38. 10640989 10.1002/(SICI)1096-9896(200001)190:1<31::AID-PATH505>3.0.CO;2-#

[30] Lothe RA , Slettan A, Sæter G, et al Alterations at chromosome 17 loci in peripheral nerve sheath tumors. J Neuropathol *Exp Neurol.* 1995;54(1):65–73. 7815081 10.1097/00005072-199501000-00008

[31] Mechtersheimer G , Otaño-Joos M, Ohl S, et al Analysis of chromosomal imbalances in sporadic and NF1-associated peripheral nerve sheath tumors by comparative genomic hybridization. Genes Chromosomes Cancer. 1999;25(4):362–369. 10398430

[32] Plaat BEC , Molenaar WM, Mastik MF, et al Computer-assisted cytogenetic analysis of 51 malignant peripheral-nerve-sheath tumors: sporadic vs. neurofibromatosis-type-1-associated malignant schwannomas. Int J Cancer. 1999;83(2):171–178. 10471523 10.1002/(sici)1097-0215(19991008)83:2<171::aid-ijc5>3.0.co;2-s

[33] Koga T , Iwasaki H, Ishiguro M, Matsuzaki A, Kikuchi M. Frequent genomic imbalances in chromosomes 17, 19, and 22q in peripheral nerve sheath tumours detected by comparative genomic hybridization analysis. J Pathol. 2002;197(1):98–107. 12081210 10.1002/path.1101

[34] Watson MA , Perry A, Tihan T, et al Gene expression profiling reveals unique molecular subtypes of neurofibromatosis type I-associated and sporadic malignant peripheral nerve sheath tumors. Brain Pathol. 2004;14(3):297–303. 15446585 10.1111/j.1750-3639.2004.tb00067.x PMC8095784

[35] Legius E , Dierick H, Wu R, et al TP53 mutations are frequent in malignant NFI tumors. Genes Chromosomes Cancer. 1994;10(4):250–255. 7522538 10.1002/gcc.2870100405

[36] Leroy K , Dumas V, Martin-Garcia N, et al Malignant peripheral nerve sheath tumors associated with neurofibromatosis type 1: a clinicopathologic and molecular study of 17 patients. Arch Dermatol. 2001;137(7):908–913. 11453810

[37] Birindelli S , Perrone F, Oggionni M, et al Rb and TP53 pathway alterations in sporadic and NF1-related malignant peripheral nerve sheath tumors. Lab Invest. 2001;81(6):833–844. 11406645 10.1038/labinvest.3780293

[38] Taubert H , Würl P, Bache M, et al The p53 gene in soft tissue sarcomas: prognostic value of DNA sequencing versus immunohistochemistry. Anticancer Res. 1998;18(1A):183–187. 9568075

[39] Watanabe T , Oda Y, Tamiya S, et al Malignant peripheral nerve sheath tumours: high Ki67 labelling index is the significant prognostic indicator. Histopathology. 2001;39(2):187–197. 11493336 10.1046/j.1365-2559.2001.01176.x

[40] Lothe RA , Smith-Sørensen B, Hektoen M, et al Biallelic inactivation of TP53 rarely contributes to the development of malignant peripheral nerve sheath tumors. Genes Chromosomes Cancer. 2001;30(2):202–206. 11135438

[41] Zou C , Smith KD, Liu J, et al Clinical, pathological, and molecular variables predictive of malignant peripheral nerve sheath tumor outcome. Ann Surg. 2009;249(6):1014–1022. 19474676 10.1097/SLA.0b013e3181a77e9a

[42] Stucky C-CH , Johnson KN, Gray RJ, et al Malignant peripheral nerve sheath tumors (MPNST): the Mayo Clinic experience. Ann Surg Oncol. 2012;19(3):878–885. 21861229 10.1245/s10434-011-1978-7

[43] Ron E , Modan B, Boice JD Jr, et al Tumors of the brain and nervous system after radiotherapy in childhood. N Engl J Med. 1988;319(16):1033–1039. 3173432 10.1056/NEJM198810203191601

[44] Evans DGR , Birch JM, Ramsden RT, Sharif S, Baser ME. Malignant transformation and new primary tumours after therapeutic radiation for benign disease: substantial risks in certain tumour prone syndromes. J Med Genet. 2006;43(4):289–294. 16155191 10.1136/jmg.2005.036319 PMC2563223

[45] Evans DGR , Baser ME, McGaughran J, et al Malignant peripheral nerve sheath tumours in neurofibromatosis 1. J Med Genet. 2002;39(5):311–314. 12011145 10.1136/jmg.39.5.311 PMC1735122

[46] Yamanaka R , Hayano A. Radiation-induced malignant peripheral nerve sheath tumors: a systematic review. World Neurosurg. 2017;105(105):961–970.e8. 28602926 10.1016/j.wneu.2017.06.010

[47] Jansma CYMN , Acem I, Grünhagen DJ, Verhoef C, Martin E; MONACO Collaborators. Local recurrence in malignant peripheral nerve sheath tumours: multicentre cohort study. BJS Open. 2024;8(2):zrae024. 38620136 10.1093/bjsopen/zrae024 PMC11018273

