# Supplementary material for: The role of radiotherapy in MPNST and the impact of NF1 status on outcomes: Insights from a multicenter cohort study
Source: Neuro Oncol. 2025 Aug 9;27(12):3214–23. doi: 10.1093/neuonc/noaf186 (PMC12916730; doi:10.1093/neuonc/noaf186)
Supplement: noaf186_Supplementary_Materials_1 [file noaf186_supplementary_materials_1.docx]

| **Supplementary Table 1**. Subgroup of NF1 patients and RTx administration | | | | |
| --- | --- | --- | --- | --- |
|  | **Overall** | **No radiotherapy** | **Any type of radiotherapy** | **p-value** |
| **Variable** |  |  |  |  |
| **N** | 165 | 71 | 94 |  |
| **Age (years)** |  |  |  |  |
| Mean (SD) |  | 34.03 (16.27) | 38.26 (14.94) | 0.085 |
| **Male gender** | 85 | 35 (49.3%) | 50 (53.8%) | 0.682 |
| NA | 1 | 0 | 1 |  |
| **ASA** |  |  |  |  |
| I | 34 | 16 (44.4%) | 18 (34.6%) | 0.613 |
| II | 47 | 17 (47.2%) | 30 (57.7%) |  |
| III | 7 | 3 (8.3%) | 4 (7.7%) |  |
| NA | 77 | 35 | 42 |  |
| **Tumor size** |  |  |  |  |
| <5 cm | 25 | 12 (21.1%) | 13 (17.3%) | 0.809 |
| 5-10 cm | 64 | 26 (45.6%) | 38 (50.7%) |  |
| >10 cm | 43 | 19 (33.3%) | 24 (32.0%) |  |
| NA | 33 | 14 | 19 |  |
| **Tumor depth** |  |  |  |  |
| Superficial | 15 | 6 (15.0%) | 9 (15.0%) | 1.00 |
| Deep | 85 | 34 (85.0%) | 51 (85.0%) |  |
| NA | 65 | 31 | 34 |  |
| **Tumor grade** |  |  |  |  |
| High grade | 95 | 36 (85.0%) | 59 (95.2%) | 0.104 |
| Low grade | 10 | 7 (16.3%) | 3 (4.8%) |  |
| NA | 60 | 28 | 32 |  |
| **RTx-associated** |  |  |  |  |
| No | 163 | 71 (100.0%) | 92 (97.9%) | 0.604 |
| Yes | 2 | - | 2 (2.1%) |  |
| **Site of primary tumor** |  |  |  |  |
| Head and neck | 17 | 7 (9.9%) | 10 (10.6%) | 0.003 |
| Extremities | 77 | 23 (32.4%) | 54 (57.4%) |  |
| Central | 71 | 41 (57.7%) | 30 (31.9%) |  |
| **Surgical margin** |  |  |  |  |
| R0 | 107 | 47 (66.2%) | 60 (63.8%) | 0.909 |
| R1 | 47 | 19 (26.8%) | 28 (29.8%) |  |
| Unknown | 11 | 5 | 6 |  |

N, Number of; RTx, radiotherapy; NA, not available; NF1, neurofibromatosis type 1; SD, standard deviation; cm, centimeter


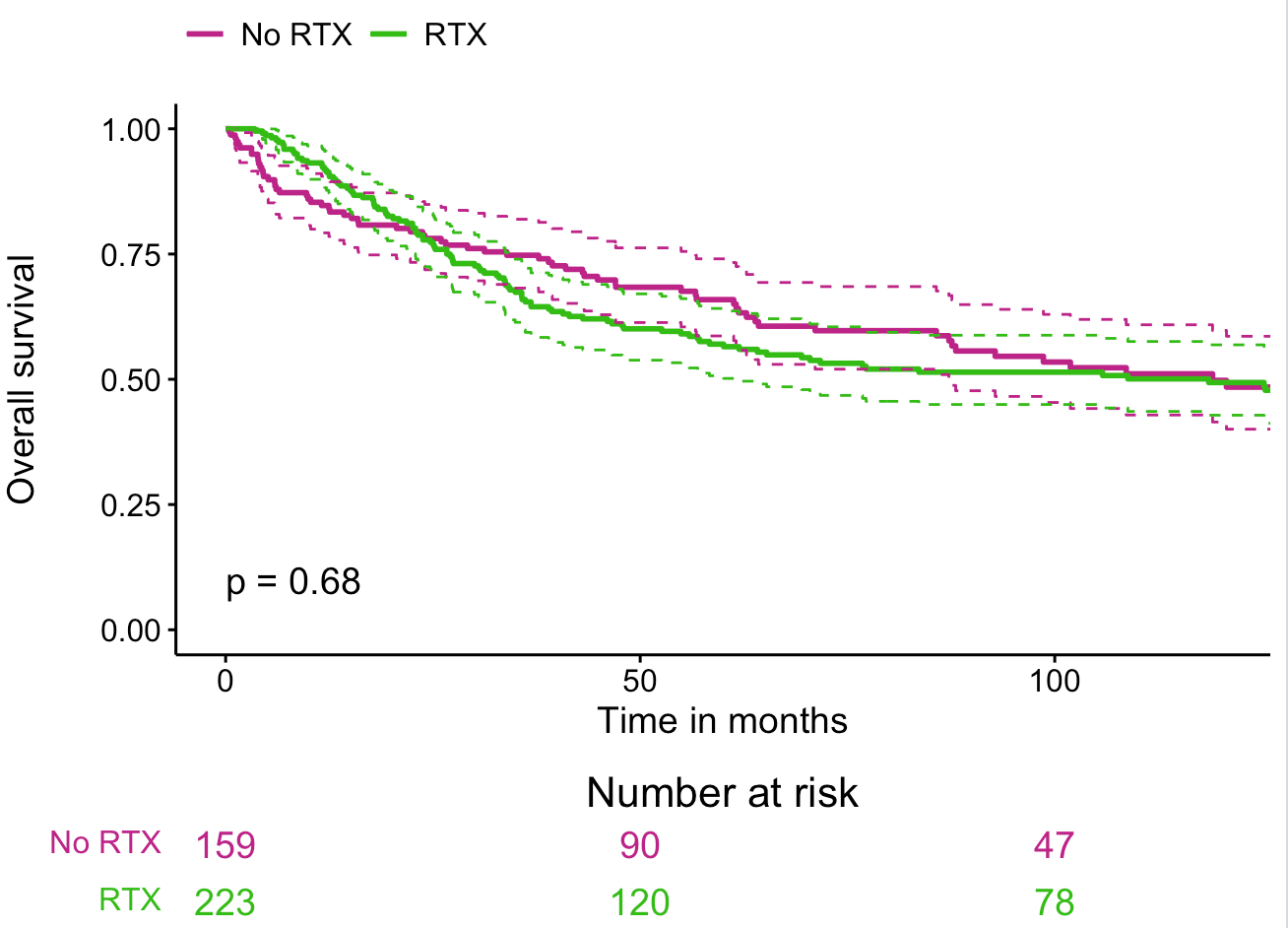


**Supplementary Figure 1**. Overall survival of subset of MPNST patients meeting WHO guidelines


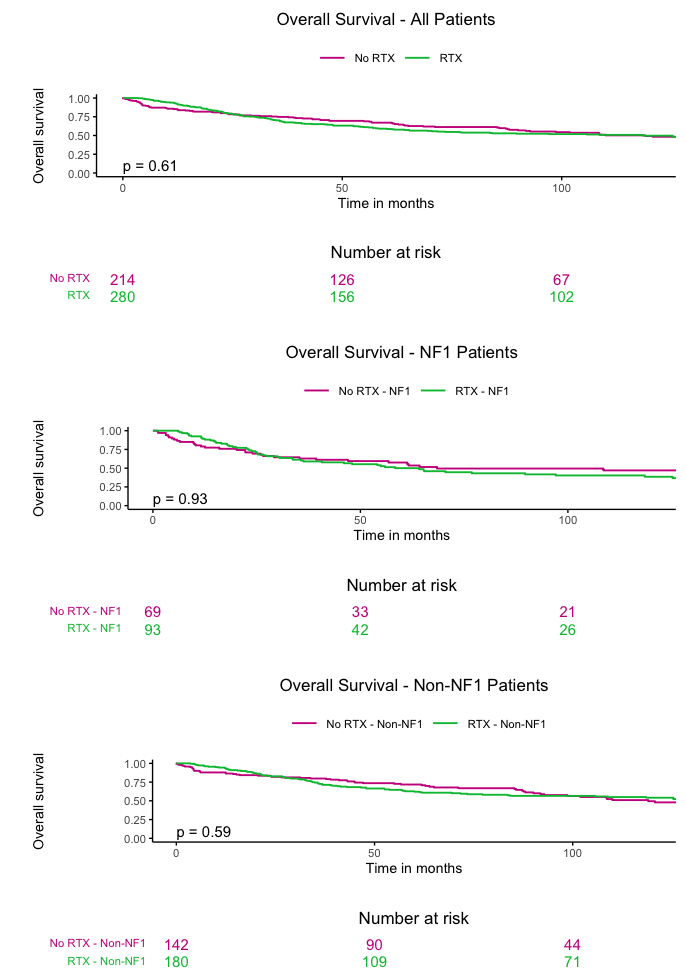


**Supplementary Figure 2**. Overall survival by RTx and NF1-status
